# Assessing the Role of Artificial Intelligence in the Creation of Patient Educational Videos for Corneal Refractive Surgery

**DOI:** 10.7759/cureus.71447

**Published:** 2024-10-14

**Authors:** Kenneth D Han, Muhammed A Jaafar, Kayvon A Moin, Phillip C Hoopes, Majid Moshirfar

**Affiliations:** 1 Ophthalmology, The University of Arizona College of Medicine - Phoenix, Phoenix, USA; 2 Ophthalmology, Hoopes Vision, Draper, USA; 3 Ophthalmology, American University of the Caribbean School of Medicine, Cupecoy, SXM; 4 Ophthalmology, John A. Moran Eye Center, University of Utah School of Medicine, Salt Lake City, USA; 5 Eye Banking and Corneal Transplantation, Utah Lions Eye Bank, Murray, USA

**Keywords:** ai, eye, generative ai, large language model, lasik, ophthalmology, patient education, prk, smile, text-to-video

## Abstract

Purpose: We aim to assess the ability of artificial intelligence (AI) to generate patient educational videos for various corneal refractive surgeries.

Methods: Three AI text-to-video platforms (InVideo (San Francisco, CA), ClipTalk (San Francisco, CA), and EasyVid (Los Angeles, CA)) were used to create patient educational videos for laser-assisted in situ keratomileusis (LASIK), photorefractive keratectomy (PRK), and small incision lenticule extraction (SMILE), respectively. Videos for LASIK and PRK from the American Academy of Ophthalmology (AAO) and a SMILE video from Zeiss served as controls for each surgery. A three-point grading system (from zero to three, with zero being the worst and three being the best in each category) was used to compare videos in terms of "image accuracy," "script accuracy," "image clarity," and "script alignment."

Results: In terms of image accuracy, the control videos outperformed InVideo, EasyVid, and ClipTalk for LASIK (3 versus 0.667 versus 0 versus 0; p<0.005), PRK (3 versus 1 versus 0.33 versus 0; p<0.05 for InVideo, p<0.005 all), and SMILE (3 versus 0.33 versus 0 versus 0.33; p<0.005), respectively. With a few exceptions, all three AI models performed similarly to the control videos in terms of script accuracy, image clarity, and script alignment.

Conclusion: In their current state, AI text-to-video generators can produce surgical educational videos for patients with accurate script narration and high image clarity, although these platforms are not yet capable of producing medically accurate images to go along with these scripts. Further improvements in the medical accuracy of these images must be made to make these videos more appropriate for patient consumption.

## Introduction

Patient preparedness for surgery varies widely based on a multitude of factors, including, but not limited to, educational level, exposure to informative content, and the extent of face-to-face counseling. Previous studies have demonstrated a clear connection between preoperative preparedness level and patient anxiety levels, postoperative satisfaction, and even surgical outcomes [1,2]. Online instructional videos represent a popular avenue through which many patients seek preoperative information and upon which they subsequently base their expectations [3].

A thorough evaluation of these educational videos, especially those found on YouTube (Google, San Bruno, CA), has been performed in the existing literature. These studies have demonstrated high variability in both the accuracy and quality of videos. While some videos prepare patients well by providing comprehensive, patient-centered explanations of surgeries and helping set appropriate expectations [4,5], others have been shown to contain medically inaccurate information, failing to adequately prepare patients for their surgeries [3,6,7].

Thus, preoperative patient educational videos represent a powerful tool that can help empower and inform patients about what to expect from their surgery. However, given the plethora of videos currently available, it may be difficult for patients to discern which videos are medically accurate. This has led some to question what role emerging technologies, such as artificial intelligence (AI), might play in the creation of these types of educational videos [8].

The recent rise in the popularity of AI, both among the public and within the medical community, is evident from numerous studies assessing its capabilities in various aspects of medicine. Thus far, AI has been implemented in charting, high-risk decision-making processes, and even robotic surgery, with varying degrees of success [9-11]. Additionally, several studies have evaluated the ability of AI models to provide feedback on videos of surgeons practicing various procedures [12,13]. Given the growing interest in AI, the objective of our study was to investigate its ability to generate accurate patient educational videos for three popular corneal refractive surgeries.

## Materials and methods

This is an observational study comparing the ability of three popular AI text-to-video platforms to create ophthalmic surgical instructional videos. The study was approved by the Biomedical Research Alliance of New York Institutional Review Board (IRB number: A20-12-547-823) and adhered to the tenets of the Declaration of Helsinki. The study was also approved by the Hoopes Vision Ethics Board.

The three AI platforms (InVideoAI (San Francisco, CA), EasyVid (Los Angeles, CA), and ClipTalk (San Francisco, CA)) were selected based on their popularity and ability to generate videos from inputted text. Videos were generated for laser-assisted in situ keratomileusis (LASIK), photorefractive keratectomy (PRK), and small incision lenticule extraction (SMILE) and were compared to control videos used as the gold standard. The control videos for LASIK and PRK included educational videos from the American Academy of Ophthalmology (AAO) (San Francisco, CA) [14,15]. The control video for SMILE was obtained from Zeiss (Oberkochen, Germany) [16].

The following prompt was developed to create a detailed, step-by-step instructional video: "Create a patient-centered educational video depicting a step-by-step guide on what to expect during LASIK, PRK, or SMILE surgery. Be as anatomically and surgically accurate as possible." This prompt was inputted into each of the AI platforms to generate instructional videos for each surgery, and the length of each video was recorded.

To objectively evaluate the quality of the AI-generated videos compared to the control videos, a four-category quality assessment was conducted, including "image accuracy" (medical accuracy of images), "script accuracy" (medical accuracy of the narrated script), "image clarity" (image resolution), and "script alignment with the video" (the script and the video progress synchronously). Each category was graded on a three-point scale, with "zero" being the worst and "three" being the best in each category (Table 1). This system was used to evaluate all control and AI-generated videos. Three independent human observers (MM, PCH, and KAM), each with experience in corneal refractive surgery, independently graded the videos according to the four-category assessment. The scores from the three observers were then averaged to create a single score for image accuracy, script accuracy, image clarity, and script alignment. The scores for each category were summed to produce a total overall score for the control, InVideo, EasyVid, and ClipTalk videos. Additionally, qualitative observations of notable findings were recorded for each platform.

**Table 1:** Grading system for instructional video quality A four-category grading system with a maximum score of three points in each category to objectively assess the quality of videos produced by the AI platforms as well as the controls Created by the authors using Microsoft Office (Microsoft Corp., Redmond, WA)

Category	Score	Description
Image accuracy	0	No images are accurate for any step of the procedure
	1	Images are accurate for some steps of the procedure
	2	Images are accurate for most steps of the procedure
	3	Images are accurate for all steps of the procedure
Script accuracy	0	No statements are accurate for any step of the procedure
	1	Statements are accurate for some steps of the procedure
	2	Statements are accurate for most steps of the procedure
	3	Statements are accurate for all steps of the procedure
Image clarity	0	No images are of high clarity and resolution
	1	Some images are of high quality and resolution
	2	Most images are of high quality and resolution
	3	All images are of high quality and resolution
Script and image alignment	0	None of the script aligns with the video
	1	Some of the script aligns with the video
	2	Most of the script aligns with the video
	3	All of the script aligns with the video

Since one AI text-to-video platform, InVideo, had the capability to modify videos based on feedback, we inputted a series of additional prompts to qualitatively assess various features of this AI model (Table 2). These included URL links to YouTube videos and a published article for use as references to improve accuracy [17,18].

**Table 2:** Follow-up prompts administered to InVideo and respective purposes These prompts were given to InVideo after its initial video creation in order to test its capabilities in reformatting the video based on feedback Created by the authors using Microsoft Office* *(Microsoft Corp., Redmond, WA)

Prompt	Capability being tested
"Be as concise as possible and only keep medically relevant images and clips from the original video"	Ability to recognize essential phrases as well as images and clips relevant to the surgery
"Incorporate clips from the following video: https://www.youtube.com/watch?v=8ot-S45d5hU"	Ability to use footage from a provided URL
"Incorporate clips using the uploaded MP4 file"	Ability to use footage from an uploaded video
"Re-write the script using the following article as a reference: https://www.ncbi.nlm.nih.gov/books/NBK555970/"	Ability to read and incorporate information from a provided article

Data analysis

Microsoft Excel version 16.0 (Microsoft Corp., Redmond, WA), IBM Statistical Package for the Social Sciences (SPSS) version 29.0 (IBM Corp., Armonk, NY), and G*Power version 3.1 (Düsseldorf, Germany) were used for all data collection and analysis. A one-way analysis of variance (ANOVA) was performed to assess for statistically significant differences in each of the quality assessment categories between the control, InVideo, EasyVid, and ClipTalk videos. The Shapiro-Wilk test was used to assess normality. Since the data were not found to be normally distributed, ANOVA tests were performed as they are considered adequate for moderate violations in the normality assumption. The statistical power of the one-way ANOVA was evaluated using G*Power software, and the effect size was calculated using Cohen's f. For our Bonferroni-corrected α value of 0.005, a medium effect size of 0.5, and four groups, a sample size of 76 videos was needed to achieve a statistical power of 0.80. Additionally, a post hoc analysis of the one-way ANOVA with our sample size of 48 scores revealed a statistical power of 0.47.

## Results

The videos created by the three AI platforms for LASIK, PRK, and SMILE are shown in Videos 1-3.

**Video 1:** Videos created for LASIK by EasyVid, ClipTalk, and InVideo LASIK: laser-assisted in situ keratomileusis

**Video 2:** Videos created for PRK by EasyVid, ClipTalk, and InVideo PRK: photorefractive keratectomy

**Video 3:** Videos created for SMILE by EasyVid, ClipTalk, and InVideo SMILE: small incision lenticule extraction

Objective assessment of AI models

**Figure 1:**
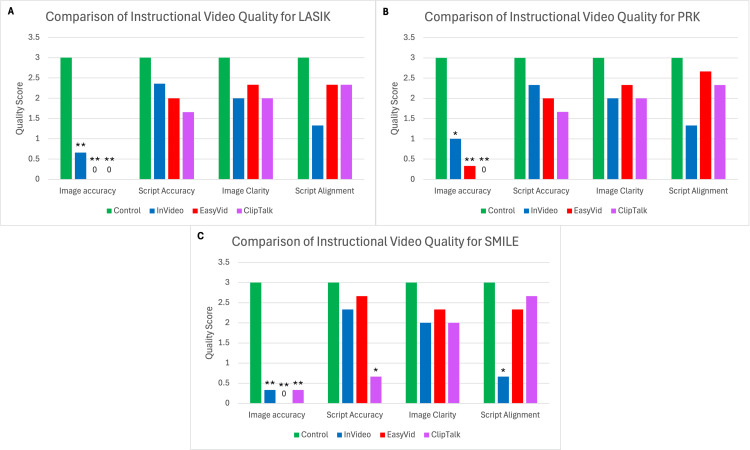
Comparison of instructional video quality for LASIK, PRK, and SMILE A: LASIK, B: PRK, C: SMILE Average observer quality score assigned to control, InVideo, EasyVid, and ClipTalk for the categories of image accuracy, script accuracy, image clarity, and script alignment Statistical significance determined with one-way ANOVA: *significant compared to control at p<0.05 and **significant compared to control at p<0.001 LASIK: laser-assisted in situ keratomileusis, PRK: photorefractive keratectomy, SMILE: small incision lenticule extraction, ANOVA: analysis of variance

When comparing the medical accuracy of images used in each instructional video, the control videos outperformed InVideo, EasyVid, and ClipTalk for LASIK (3 versus 0.667 versus 0 versus 0; p<0.005), PRK (3 versus 1 versus 0.33 versus 0; p<0.05 for InVideo, p<0.005 overall), and SMILE (3 versus 0.33 versus 0 versus 0.33; p<0.005). No significant differences in medical accuracy of images were noted between the AI platforms themselves (p>0.05). Additionally, in terms of video quality and resolution, no significant differences were found between any of the instructional videos (p>0.05) (Figure 1).

In terms of script accuracy, no significant differences were found, except for the control video having a higher script accuracy score than ClipTalk for the SMILE instructional video (3 versus 0.67; p<0.05) (Figure 1). Qualitatively, the scripts from each AI platform were more accurate than their corresponding images. Additionally, each AI platform made errors in their scripts with varying degrees of severity, although these differences were not always statistically significant compared to the control based on the grading scale used for script accuracy (p>0.05).

In addition, in terms of script alignment, the AI-generated videos had comparable alignment between the script and the images being displayed compared to the control videos, with the exception of the SMILE video generated by the control, which had better alignment than InVideo (3 versus 0.67; p<0.05) (Figure 1).

**Figure 2:**
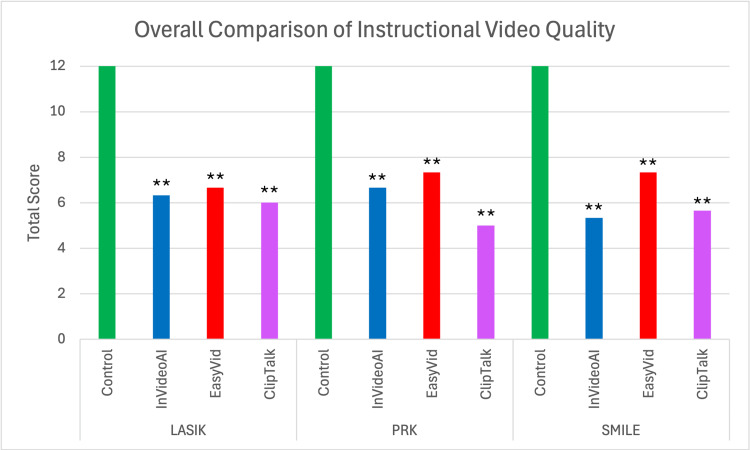
Overall comparison of instructional video quality Comparison of the total quality score assigned to instructional videos created by the control, InVideo, EasyVid, and ClipTalk for LASIK, PRK, and SMILE Statistical significance determined with one-way ANOVA: **significant compared to control at p<0.001 LASIK: laser-assisted in situ keratomileusis, PRK: photorefractive keratectomy, SMILE: small incision lenticule extraction, ANOVA: analysis of variance

After calculating a total score for each video platform, the control videos were found to outperform all three AI text-to-video generators for LASIK (12 (control) versus 6.33 (InVideo) versus 6.67 (EasyVid) versus 6 (ClipTalk); p<0.005), PRK (12 versus 6.67 versus 7.33 versus 5; p<0.005), and SMILE (12 versus 5.33 versus 7.33 versus 5.67; p<0.005) (Figure 2).

**Figure 3:**
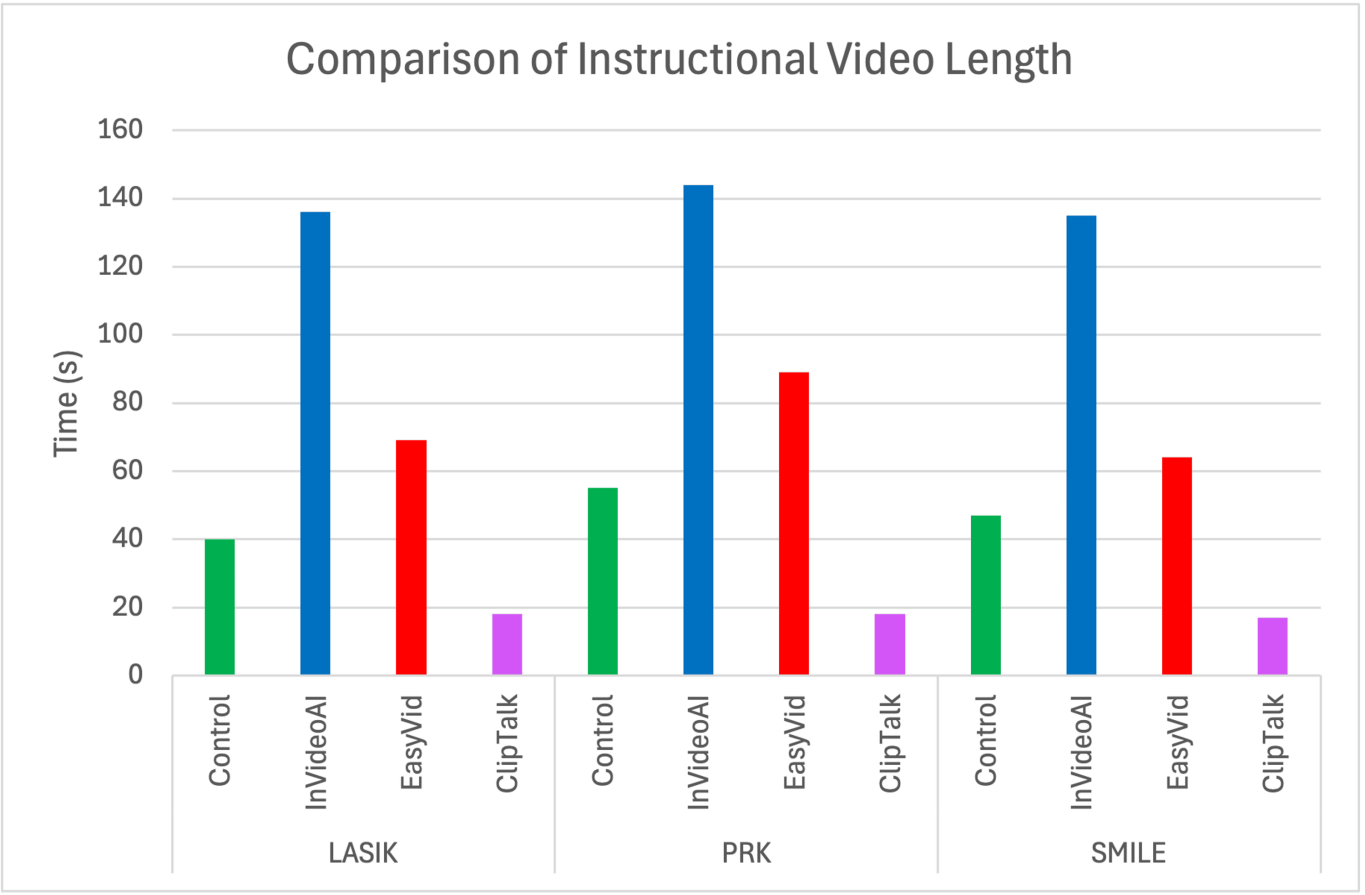
Comparison of instructional video length The length of videos generated by the control, InVideo, EasyVid, and ClipTalk in seconds for LASIK, PRK, and SMILE No statistical tests were used for this figure. LASIK: laser-assisted in situ keratomileusis, PRK: photorefractive keratectomy, SMILE: small incision lenticule extraction

In terms of the length of patient educational videos, InVideo used a combination of stock videos and images to create the longest videos for LASIK, PRK, and SMILE (136, 144, and 135 seconds, respectively). This was followed by EasyVid (69, 89, and 64 seconds), control videos (40, 55, and 47 seconds), and ClipTalk (18, 18, and 17 seconds) in order from longest to shortest (Figure 3). Notably, ClipTalk and EasyVid both utilized AI-generated images rather than stock videos or clips.

Qualitative findings

One of the AI text-to-video platforms included in this study, InVideo, had the ability to receive feedback and make video edits. To explore this feature, follow-up prompts were developed to test various capabilities of the AI model (Table 2). When administering follow-up prompts to InVideo to create a more concise script, the platform made minor changes, such as removing phrases such as "buckle up and let's dive right in," while retaining the main steps of the surgery. However, it was unable to eliminate irrelevant video clips, stating that this was outside of its capabilities. Additionally, when follow-up prompts were used to test the platform's ability to recognize anatomical structures, incorporate aspects of a provided video URL, or use clips from an uploaded MP4 file, InVideo refused to follow these prompts and did not adjust the video clips accordingly. However, when given a URL to an article containing information about LASIK surgery, the AI platform was able to incorporate phrases such as "place an eyelid speculum" and "administer antibiotic and anti-inflammatory drops to help prevent infection and inflammation" into its script.

Furthermore, certain patterns were recognized regarding the types of inaccuracies of the images provided by each platform. InVideo had a tendency to use a combination of non-surgical ophthalmic video clips, including slit lamp examinations, as well as seemingly random clips from different surgical specialties. On the other hand, EasyVid and ClipTalk almost always displayed AI-generated images that were related to ophthalmic surgery but included completely factitious details such as a laser emitting directly from an overhead surgical spotlight.

## Discussion

Patients often seek out instructional videos prior to surgery to help them prepare and set expectations. Our study investigated the capabilities of text-to-video AI platforms in creating patient educational videos for various corneal refractive surgeries.

The video clips used in the control videos were more medically accurate than those in the AI-generated videos for all three surgeries evaluated (Figure 1). InVideo was the only platform that incorporated video clips and managed to display some accurate visuals, such as slit lamp examinations, patient positioning for corneal refractive surgery, and the administration of eye drops. However, InVideo was largely limited by its use of stock photos and videos from a database that did not appear to contain any actual images of corneal refractive surgery. In contrast, EasyVid and ClipTalk created shorter videos using AI-generated images and a voice-over in a slideshow format, rather than stock footage. The images generated by these two platforms were tangentially related to corneal refractive surgery but were highly inaccurate and contained unrealistic visuals. This finding aligns with previous studies demonstrating a low degree of accuracy in AI-generated medical images [19]. If provided with a database of stock images containing accurate corneal refractive surgery footage, platforms such as InVideo would come closest to producing accurate educational surgical videos for patients. Therefore, further advancements are necessary regarding the ability of AI models to incorporate video clips from more specialized medical databases, as this could help overcome the use of unrelated clips from other surgical specialties as well as inaccurate AI-generated images in the creation of these videos.

In contrast to the poor medical accuracy of images used in these AI-generated videos, all three AI platforms performed well in generating accurate scripts (Figure 1). None of the AI-generated videos had significantly poorer script accuracy than the control videos, except for the SMILE instructional video created by ClipTalk. These findings are consistent with previous studies that have demonstrated AI models' ability to generate accurate medical information, although with certain limitations [20,21]. With future improvements in other aspects of text-to-video AI models, these medically accurate scripts may play a powerful role in the creation of patient instructional videos.

No significant differences in image clarity were found between any of the AI platforms and the control videos (Figure 1). All video platforms evaluated in this study, including the control, offered adjustable image resolutions up to at least 720p. These findings are consistent with previous studies that have demonstrated AI's ability to produce high-clarity images [22]. Therefore, when it comes to producing AI-generated surgical patient educational videos, image clarity is not the limiting factor.

For the most part, AI-generated surgical educational videos were able to align their scripts with the images and video clips being displayed (Figure 1). Previous studies in radiology have demonstrated AI's remarkable ability to interpret medical images [23]. This suggests that these platforms may possess some capability to recognize the content being displayed and match it with the appropriate verbiage, helping patients understand the steps of surgery.

Additionally, in some aspects, the AI platforms performed better than the control videos used in our study. For example, one platform provided more detailed perioperative information, such as mentioning that the patient would be "lying down comfortably" and would receive anesthetic eye drops to "ensure that the entire procedure (would be) painless." Such details can be valuable to patients but were not included in the control videos, which focused more on the specific steps of the surgery.

One limitation of this study includes the use of a limited number of text-to-video AI platforms. While there are several other popular AI text-to-video platforms currently available, each had specific limitations that precluded their inclusion in our study, which resulted in the limitation of only analyzing three AI text-to-video platforms in our study. For instance, Canva (Sydney, Australia) creates videos that last only a few seconds. Deep Brain (Palo Alto, California) generates "talking head" videos, in which an AI-generated reporter reads a provided script. Similarly, Pictory (Bothell, Washington) relies on a provided script and does not generate its own based on a prompt. Additionally, the grading system used in this study has not been validated, and further research is needed to assess its validity. For example, it is unclear whether the grading system used may have been biased toward one AI platform that happened to fit our criteria better than the others. Additionally, the grading system in our study did not include some details that might be important to patients such as video length. Lastly, the grading system was mainly used to assess the medical accuracy of these videos and did not highlight other parts of these clips that are more tailored toward patient comfort. However, to our knowledge, this is the first study of its kind to assess AI as an emerging technology in the creation of patient instructional videos for various corneal refractive surgeries.

## Conclusions

AI text-to-video generators are a rapidly advancing technology with vast potential in the field of medicine. Our study evaluated the ability of these platforms to create surgical educational videos for patients and found that the script accuracy, script alignment, and image clarity used in these videos largely performed well. However, the images and clips lacked medical accuracy, and no platform was able to fully and accurately depict corneal refractive surgery. With further advancements in AI and its ability to access and incorporate medical image databases, it is hoped that AI-based text-to-video generators will one day assist in preparing patients for corneal refractive surgery. Once these advancements are made, future studies should be performed to assess these updated capabilities. However, in their current state, these platforms are not able to produce educational videos with sufficiently accurate medical images for patient consumption.

## References

[1] Jones BA, Richman J, Rubyan M (2024). Preoperative education is associated with adherence to downstream components and outcomes in a colorectal surgery enhanced recovery program. Ann Surg Open.

[2] Khorfan R, Shallcross ML, Yu B (2020). Preoperative patient education and patient preparedness are associated with less postoperative use of opioids. Surgery.

[3] Javidan A, Nelms MW, Li A, Lee Y, Zhou F, Kayssi A, Naji F (2023). Evaluating YouTube as a source of education for patients undergoing surgery: a systematic review. Ann Surg.

[4] Monteiro Grilo A, Ferreira AC, Pedro Ramos M, Carolino E, Filipa Pires A, Vieira L (2022). Effectiveness of educational videos on patient's preparation for diagnostic procedures: systematic review and meta-analysis. Prev Med Rep.

[5] Zheng Y, Yan Q (2023). Effect of application of short-form video health education on the health knowledge and satisfaction with nursing care of patients with lower extremity fractures. BMC Nurs.

[6] Hohenleitner J, Barron K, Bostonian T, Demyan L, Bonne S (2023). Educational quality of YouTube videos for patients undergoing elective procedures. J Surg Res.

[7] Starks C, Akkera M, Shalaby M (2021). Evaluation of YouTube videos as a patient education source for novel surgical techniques in thyroid surgery. Gland Surg.

[8] Mohamed AA, Lucke-Wold B (2024). Text-to-video generative artificial intelligence: sora in neurosurgery. Neurosurg Rev.

[9] Knudsen JE, Ghaffar U, Ma R, Hung AJ (2024). Clinical applications of artificial intelligence in robotic surgery. J Robot Surg.

[10] Nashwan AJ, Abujaber AA (2023). Harnessing large language models in nursing care planning: opportunities, challenges, and ethical considerations. Cureus.

[11] Bahl M, Barzilay R, Yedidia AB, Locascio NJ, Yu L, Lehman CD (2018). High-risk breast lesions: a machine learning model to predict pathologic upgrade and reduce unnecessary surgical excision. Radiology.

[12] Pugh CM, Ghazi A, Stefanidis D, Schwaitzberg SD, Martino MA, Levy JS (2020). How wearable technology can facilitate AI analysis of surgical videos. Ann Surg Open.

[13] Goodman ED, Patel KK, Zhang Y (2024). Analyzing surgical technique in diverse open surgical videos with multitask machine learning. JAMA Surg.

[14] (2024). LASIK (microkeratome). https://www.youtube.com/watch?v=ynCwYgKcrZQ.

[15] (2024). What is photorefractive keratectomy (PRK)?. https://www.youtube.com/watch?v=EoWdquQfAbA.

[16] (2024). SMILE(small incision lenticule extraction). https://www.youtube.com/watch?v=wW4W7QqfzLo.

[17] (2024). LASIK (laser in situ keratomileusis). https://www.youtube.com/watch?v=8ot-S45d5hU.

[18] Moshirfar M, Bennett P, Ronquillo Y (2024). Laser in situ keratomileusis (LASIK). https://pubmed.ncbi.nlm.nih.gov/32310430/.

[19] Moin KA, Nasir AA, Petroff DJ, Loveless BA, Moshirfar OA, Hoopes PC, Moshirfar M (2024). Assessment of generative artificial intelligence (AI) models in creating medical illustrations for various corneal transplant procedures. Cureus.

[20] Johnson D, Goodman R, Patrinely J (2023). Assessing the accuracy and reliability of AI-generated medical responses: an evaluation of the chat-GPT model. Res Sq.

[21] Jongsma KR, Sand M, Milota M (2024). Why we should not mistake accuracy of medical AI for efficiency. NPJ Digit Med.

[22] Pinto-Coelho L (2023). How artificial intelligence is shaping medical imaging technology: a survey of innovations and applications. Bioengineering (Basel).

[23] Saha A, Bosma JS, Twilt JJ (2024). Artificial intelligence and radiologists in prostate cancer detection on MRI (PI-CAI): an international, paired, non-inferiority, confirmatory study. Lancet Oncol.

